# Recent Advances in Electrospun Nanofiber Interfaces for Biosensing Devices

**DOI:** 10.3390/s17081887

**Published:** 2017-08-16

**Authors:** Eleni Sapountzi, Mohamed Braiek, Jean-François Chateaux, Nicole Jaffrezic-Renault, Florence Lagarde

**Affiliations:** 1 Université Lyon, CNRS, Université Claude Bernard Lyon 1, ENS de Lyon, Institute of Analytical Sciences, UMR 5280, 5 Rue la Doua, F-69100 Villeurbanne, France; elena.sapountzi@gmail.com (E.S.); mohamed_braiek@yahoo.fr (M.B.); nicole.jaffrezic@isa-lyon.fr (N.J.-R.); 2 Laboratoire des Interfaces et des Matériaux Avancés, Faculté des Sciences de Monastir, Avenue de l’Environnement, University of Monastir, Monastir 5019, Tunisia; 3 Université Lyon, Université Claude Bernard Lyon 1, CNRS, Institut des Nanotechnologies de Lyon, UMR5270, Bâtiment Léon Brillouin, 6, rue Ada Byron, F-69622 Villeurbanne CEDEX, France; jean-francois.chateaux@univ-lyon1.fr

**Keywords:** electrospinning, biosensing devices, bioreceptor immobilization, carbon nanofibers, metal oxide nanofibers, polymer nanofibers, metal nanoparticles, carbon nanotubes

## Abstract

Electrospinning has emerged as a very powerful method combining efficiency, versatility and low cost to elaborate scalable ordered and complex nanofibrous assemblies from a rich variety of polymers. Electrospun nanofibers have demonstrated high potential for a wide spectrum of applications, including drug delivery, tissue engineering, energy conversion and storage, or physical and chemical sensors. The number of works related to biosensing devices integrating electrospun nanofibers has also increased substantially over the last decade. This review provides an overview of the current research activities and new trends in the field. Retaining the bioreceptor functionality is one of the main challenges associated with the production of nanofiber-based biosensing interfaces. The bioreceptors can be immobilized using various strategies, depending on the physical and chemical characteristics of both bioreceptors and nanofiber scaffolds, and on their interfacial interactions. The production of nanobiocomposites constituted by carbon, metal oxide or polymer electrospun nanofibers integrating bioreceptors and conductive nanomaterials (e.g., carbon nanotubes, metal nanoparticles) has been one of the major trends in the last few years. The use of electrospun nanofibers in ELISA-type bioassays, lab-on-a-chip and paper-based point-of-care devices is also highly promising. After a short and general description of electrospinning process, the different strategies to produce electrospun nanofiber biosensing interfaces are discussed.

## 1. Introduction

Recent advances in nanoscience and nanotechnology have opened up new horizons for the development of biosensors of enhanced sensitivity, specificity, detection time, and low cost. Sensors miniaturization provides great versatility for incorporation into multiplexed, portable, wearable, and even implantable medical devices [1,2,3,4]. Engineering of the transducer surface using nanomaterials (i.e., nano-sized objects or nanoengineered/nanostructured materials) brings novel and sometimes unique properties that have been extensively harnessed in the past few years and addressed in several recent reviews [5,6,7,8,9]. The nanostructures used in the construction of biosensor devices vary in size (1 to 100 nm in at least one of their dimensions), shape (nanoparticles, nanotubes, nanorods, nanowires, nanofibers, nanosheets…), chemical nature (carbon-based materials, metals, metal oxides, polymers…) and physicochemical properties (electronic, optical, magnetic, mechanical, thermal…). The attractiveness of such nanomaterials relies not only on their ability to act as efficient and stabilizing platforms for the biosensing elements, but also on their small size, large surface area, high reactivity, controlled morphology and structure, biocompatibility, and in some cases electrocatalytic properties. Structuring the transducer at the nanoscale contributes to enlarge the overall surface available for bioreceptor immobilization. Incorporation of nanomaterials into the sensing layers is also often associated with higher mass transfer rates, acceleration and magnification of the transduction process, contributing to signal amplification and faster biosensor response.

Among the variety of nanostructuring materials, nanofibers (NFs) produced by electrospinning have been the object of growing interest during the past decade and the technique has been extensively reviewed with respect to its setup, mechanism, applications, advantages, technical issues, and prospective developments [10,11,12,13,14,15,16,17,18,19,20,21,22,23,24,25,26,27]. Electrospinning is a convenient and powerful technique to generate uniform sub-micron fibers in a continuous process and at large scale from a rich variety of polymers. Fiber mats, exhibiting large surface areas, may be produced on a support or used as self-standing substrates [10,11,12]. Compared to the techniques commonly used for fibers production (i.e., drawing, phase separation, template assisted or self-assembly techniques), electrospinning combines simplicity, versatility and low cost with superior capabilities to elaborate scalable ordered and complex nanofibrous assemblies [13]. Electrospun NFs have demonstrated high potential for a wide spectrum of applications such as drug delivery [13,14,15,16], tissue engineering [13,14,15,17], water treatment [18], energy conversion and storage [19,20], or electronics [21]. Due to their large surface areas, high porosity and their ability to be easily functionalized, nanofiber mats produced by electrospinning have also been increasingly exploited to enhance performances of analytical devices. Many papers have been recently dedicated to the various and exciting potentialities existing in the field. NFs can be used as sorbents in microextraction techniques [22], as chromatographic phases [23], as separators, concentrators or mixers in microfluidic systems [24,25,26]. A large number of physical and chemical sensors based on electrospun NFs have been reported, mostly for gas sensing [27,28]. The number of works related to the fabrication of electrospun NFs-based biosensing devices is also growing fast. One of the critical points to achieve efficient biosensing devices, regardless of their type (biosensors or bioassays, static of flow systems) and of the nature of biosensing elements (enzymes, antibodies…), is to elaborate functional NFs biointerfaces. This means biointerfaces where the bioreceptors’ activity and accessibility, binding events, and signal transductions are optimal. The optimization should be performed considering the analytical performances targeted, some of them (e.g., accuracy, sensitivity, selectivity) being often prioritized compared to others (e.g., response time, long-term stability).

Herein, we intend to provide the reader with a comprehensive overview of the application of electrospun NFs to the biosensing area, reporting the more recent contributions and advances in the field. Very few reviews have been dedicated to the topic [29,30,31,32] and none of them provide a complete survey, focusing more on some specific materials used to produce NFs scaffolds (e.g., metal oxides [29], nanomaterial/polymer composites [30]), the formation of biomolecules/NFs biocomposites [31], or the detection of specific target analytes (e.g., glucose [32]). After a short and general description of electrospinning process, the different strategies used in the elaboration of electrospun NFs biointerfaces for biosensor applications will be discussed. Then, recent developments in the area of ELISA-type bioassays and point-of-care devices, with a special attention to lab-on-a chip and paper-based systems, will be addressed. All kinds of bioreceptor and transduction mode, immobilization strategy, NFs material and field of application will be considered.

## 2. General Overview of Electrospinning Process

**Figure 1:**
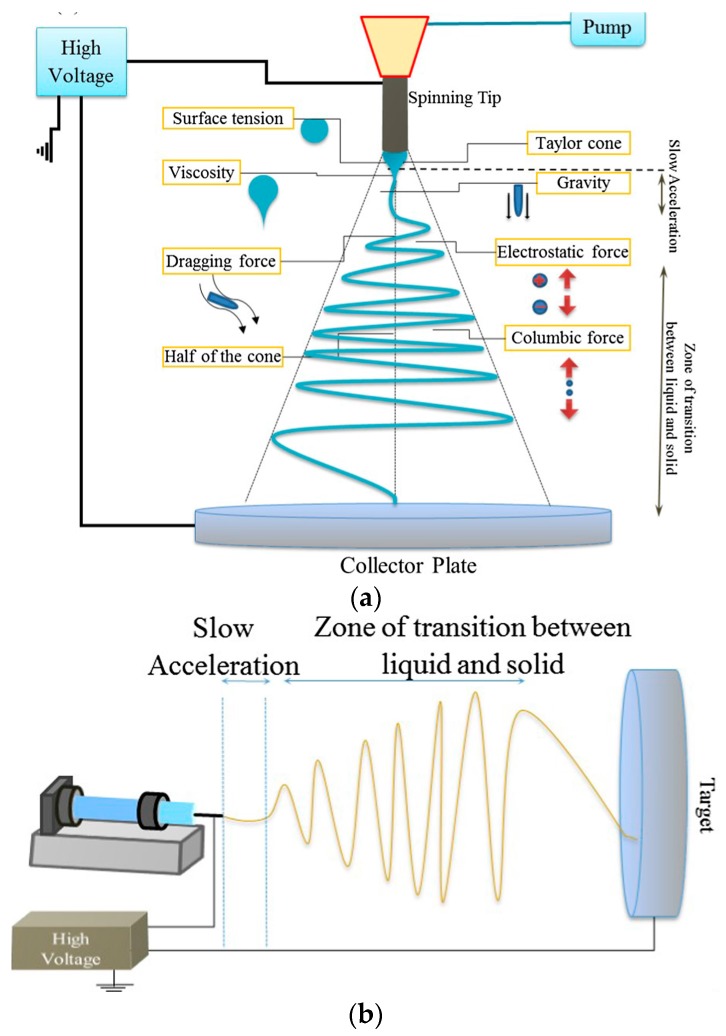
Typical horizontal (**a**) and vertical (**b**) electrospinning set-ups. They are represented with a static collector plate but other configurations exist. Reprinted with permission from [16]. Copyright 2016 Elsevier.

Electrospinning is an electrostatically-driven process ideally conducted under controlled temperature and humidity conditions [10]. Two standard set-ups, i.e., vertical or horizontal, are currently available to produce single fibers or NF mats. Both consist of four major components: a high-voltage power supply, a spinneret with a metallic needle, a syringe pump and a grounded collector (Figure 1). A polymer melt or solution is dispensed at a constant and controlled rate through the spinneret into a high voltage electrical field generated between the end of the needle and the collector. The droplet formed at the end of the needle tip, subjected to the electrical field, is first elongated into a conical shape termed as the Taylor cone. When the repulsive electrical forces overcome the surface tension forces, an electrified polymer jet ejects from the apex of the cone. The jet is then elongated and whipped continuously by electrostatic repulsion forces. Thinning of the jet results in solvent evaporation or solidification of the melt, and the deposition of a nonwoven web of solid NFs on the collector. The electrospinning process is therefore governed by a variety of forces, e.g., the Coulomb force between charges on the jet surface, the electrostatic force due to the external electric field, the viscoelastic force of the solution, the surface tension, the gravitational force (Figure 1b). The technique is highly versatile since, besides the conventional non-oriented fibers, a variety of morphologies and structures (e.g., aligned or crossed fiber arrays, ribbon, necklace-like, nanowebs, hollow, helical or coil fibers…) can be obtained by modifying electrospinning conditions or set-up (e.g., collector configuration) [33].

**Figure 2:**
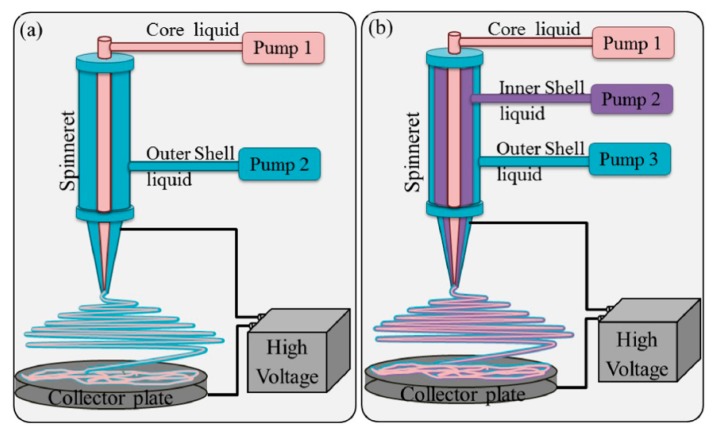
Coaxial (**a**) and triaxial (**b**) electrospinning set-ups. Reprinted with permission from [16]. Copyright 2016 Elsevier.

For specific applications, e.g., drug delivery or biosensing, where bioactive molecules should be entrapped in the fibers but should not be in direct contact with organic solvents, core-shell type NFs can be produced using modified spinneret/pump configurations, as shown in Figure 2 [16]. The thickness of the sheath and the size of the inner part of the fiber can be easily controlled by tuning the ratio of inner-outer injection speed during electrospinning.

NFs’ diameter and morphology can be controlled by a proper selection of a variety of parameters related to spinning process (i.e., applied voltage, flow-rate, collector type, needle diameter, tip-to-collector distance), polymer solution (i.e., concentration and molecular weight of the polymer, solution viscosity, surface tension, conductivity), and ambient conditions (humidity, temperature, pressure, type of atmosphere). Generally speaking, NFs’ diameter increases with increasing viscosity and polymer concentration and with decreasing conductivity, while fiber beading can be avoided in rising viscosity or using polymers with higher molecular weight. High surface tension favors jet instability. As regards as processing parameters, high voltage and low feed rate tend to decrease NFs diameter, while fiber beading is observed when the tip-to-collector distance and the polymer solution feed rate are not sufficient. Humidity and temperature conditions are also of uppermost importance, since excess of humidity results in pores formation and too high temperature leads to decreased fibers diameter [10,13].

Electrospun NFs may be fabricated from a remarkably wide range of polymers, either natural (e.g., silk fibroin, collagen, chitosan, gelatin…) or synthetic (e.g., polyacrylonitrile (PAN), polystyrene (PS), polyvinylalcohol (PVA), polyvinylpyrrolidone (PVP), polyethylenimine (PEI)…) incorporating or not fillers such as metal salts, metal nanoparticles (NPs), carbon nanotubes (CNTs), graphene, fluorescent or photoluminescent markers to provide additional advanced functionalities [28,30,34].

## 3. Electrospun NFs in Biosensors

Electrospun NF-based biosensors reported in the literature mostly rely on electrochemical transduction. Sensing elements are principally enzymes, antibodies, more scarcely DNA strands or aptamers.

The bioreceptors can be immobilized using various strategies, depending on the physical and chemical characteristics of both the recognition elements and the NF scaffolds, and on their interfacial interactions. The most common methods proposed to generate bioreceptor-NF hybrid assemblies consist in the attachment of the biomolecules onto the fiber surface by physical or chemical sorption, covalent binding, cross-linking or entrapment in a membrane. This approach has been extensively used to immobilize enzymes [35,36,37,38,39,40,41,42,43,44,45,46,47,48,49,50,51,52,53,54,55,56,57,58,59,60,61], antibodies [62,63,64,65,66,67,68,69,70], DNA strands [71,72,73] and aptamers [74,75]. Another way to proceed, more specifically developed for enzyme biosensors, consists in entrapping the bioactive molecules inside the NFs by electrospinning a blend of enzymes and polymer [76,77,78,79,80,81,82,83,84,85].

Retaining the bioreceptor functionality is one of the main challenges associated with the production of NF-based biosensing interfaces. An important requirement for the immobilization step is that the matrix provide a favorable and inert environment for the biomolecules, i.e., it should not induce severe modifications in their native structure, which would compromise their biological activity (recognition capacities, reactivity, and/or selectivity). From that point-of-view, hydrophilic polymers such as PVA or PEI are particularly well-suited and have been extensively used in the entrapment immobilization strategy. However, the resulting water-soluble NFs must be treated to guarantee further operational stability of the biosensor in aqueous media. This is generally done by cross-linking using liquid or gaseous glutaraldehyde (GA). The operational stability issue can also be solved by generating NFs of low water solubility (hydrophic polymers, carbon, or metal oxide NFs) further used to attach the bioreceptors. In this case, a post-treatment of the fibers is generally performed to improve the biocompatibility of the surface and introduce functional groups if needed.

To obtain highly sensitive biosensors, the fiber mats produced by electrospinning should also provide a large active surface area. This means that high bioreceptor loadings are requested and that the biomolecules should, not only keep their biological functionality, but also remain accessible to the molecules to be detected.

To achieve highly effective biosensors, it is finally very important that the biorecognition processes occurring at biointerfaces should be efficiently transduced into measurable signals. Different strategies have been proposed, e.g., the production of NFs doped with conducting NMs for electrochemical biosensors. Electrospinning process enables for the homogeneous distribution of nanoobjects (NPs, CNTs…) inside the fibers, resulting in enhanced electron transfers from the recognition sites to the electrodes.

### 3.1. Attachment of Sensing Biomolecules onto Electrospun NFs

NFs fabricated from a variety of materials, including metal oxides, carbon, and polymers doped or not with conductive nanomaterials (e.g., metal NPs, CNTs) or covered with a conductive polymer layer, have been explored as functional platforms for immobilizing the biosensing molecules. A summary of the enzymatic biosensors prepared this way is given in Table 1. As shown in the table, the different immobilization strategies, i.e., covalent binding, cross-linking, sorption, and entrapment in a membrane, have been used. Due to its simplicity and versatility, adsorption approach is the most commonly employed, but entrapment and covalent strategies may be preferred to avoid leaching issues. It has been shown, in many cases, that appropriate grafting of the biomolecules onto surfaces can prevent molecular movements that typically lead to conformational changes and enzymes inactivation [86].

#### 3.1.1. Metal-Oxide NFs

New developments in metal-oxide NFs-based electrochemical biosensors have been discussed in a recent review [29]. Metal-oxide NFs can be produced by electrospinning a solution containing an inorganic precursor (metal alkoxide or metal salt) and a sacrificial polymer carrier. PVP is one of the most current polymers used for this purpose. The as-spun inorganic/organic composite is further calcinated at high temperature to remove the polymer and oxidize the precursor to produce the metal-oxide phase by nucleation and growth. The fabrication of metal-oxide NFs is more complicated than the production of polymer NFs. Apart from solvent evaporation, reactions such as hydrolysis, condensation and gelation of the inorganic precursors occur once the liquid jet is ejected from the needle. If gelation process is too rapid, clogging effects are observed, and the jet becomes less elastic, preventing the electrospinning process from running properly. Controlling all the solution and process parameters precisely (i.e., type and concentration of the precursor, concentration of the polymer host, incorporation of suitable additives) is therefore essential [87]. During calcination, the polymers decompose while the inorganic precursors oxidize and crystallize forming nanocrystals aligned along what used to be the as-spun fibers. The resulting polycrystalline metal-oxide NFs exhibit unique morphologies with large surface areas, nanopores coexisting with larger pores in the structure. As such, they possess enhanced mass transfer capacities and improved electronic and optical properties compared to thin films of the same nature. Moreover, the metal-oxide NFs can be collected using a metal-frame collector to obtain aligned configurations, which facilitates the production of transistor based biosensors.

Due to the removal of the polymer and sintering of the metal oxide phase during calcination, shrinkage in NF diameter is observed, accompanied by thermal and internal mechanical stress, resulting in brittle NFs. The poor mechanical strength of metal-oxide NFs might impede the long-term stability of derived biosensors, but this issue can be solved by incorporating suitable additives in the electrospinning solution or to the NFs before heating [29].

ZnO NFs-based biosensors have been produced for the detection of various target analytes of biomedical interest. For example, Ahmad et al. [35] reported the successful fabrication of ZnO NFs from PVP and Zn acetate for amperometric glucose determination. After calcination, the NFs were transferred onto a gold electrode and covered with a PVA film to ensure their firm attachment to the electrode. Glucose oxidase (GOx) was further immobilized on the surface through physical adsorption. The biosensor response to glucose injection was very rapid (4 s) and a linear range from 0.25 to 19 mM with a low limit of detection (LOD) of 1 μM could be achieved. Immunosensors based on ZnO NFs prepared from PAN/Zn acetate blends have been also developed for the electrochemical detection of histidine-rich protein-2, synthesized and released into the blood stream by the most lethal and prominent malarial parasite *Plasmodium falciparum* [62], epidermal growth factor receptor 2ErbB-2, a breast cancer biomarker [63] and carcinoma antigen-125, an ovarian cancer biomarker [64]. To decrease the intrinsic resistivity of ZnO, and therefore improve the biosensor sensitivity, it was proposed to dope the NFs with Cu [62] or multiwall carbon nanotubes (MWCNTs) [64] by incorporating copper nitrate or MWCNTs in the electrospinning solution. Reactive carboxyl groups were generated on the NF surface by oxygen plasma treatment [64], thermal oxidation of MWCNTs [62], or by assembling mercaptopropionic acid on ZnO via interaction of hydrosulphide groups (HS^−^) with Zn^2+^ [63]. These groups were subsequently used to conjugate antibodies with the NF platform via the amino groups of the proteins using the well-established EDC/NHS coupling reaction.

In a similar way, TiO_2_, Mn_2_O_3_ and IrO_x_ NFs produced by electrospinning have been reported as very promising interfaces for biomolecules immobilization and successfully used for the elaboration of ultrasensitive biosensors. The TiO_2_ NFs were prepared from PVP and Ti butoxide or propoxide [36,37,66]. Tang et al. [36] modified Pt electrodes with TiO_2_ electrospun fiber mats and adsorbed GOx on them by drop coating. They proposed to improve immobilization by adding a chitosan film (Chit) on the GOx/TiO_2_ NFs/Pt electrodes. The addition of the TiO_2_ NFs helped increasing the biosensor response towards glucose by a factor 2.7. The Chit/GOx/TiO_2_ NFs/Pt electrode response to 100 µM glucose was respectively 4.6 and 74 times higher than those of Chit/GOx/Pt and Chit/GOx/TiO_2_ film/Pt, demonstrating the positive effect of NFs structuring. The same authors investigated the influence of TiO_2_ NFs density on the biosensor signal and demonstrated that there was an optimal value. The quantity of TiO_2_ NFs had to be sufficient to guarantee the immobilization of a large amount of GOx molecules, but too high densities resulted in a strong electron transfer resistance at the interface. More recently, Mondal et al. [37] produced partially aligned mesoporous TiO_2_ NFs at the surface of an indium tin oxide (ITO) electrode. Two enzymes, cholesterol oxidase and cholesterol esterase, were immobilized covalently to the NFs previously treated by oxygen plasma. Esterified cholesterol could be successfully detected by cyclic voltammetry with a low limit of detection (0.49 mM) and response time (20 s). In another study, a cell capture immunoassay based on electrospun TiO_2_ NF mats was proposed and applied to the detection of circulating tumor cells from colorectal and gastric cancer patients [65].

Mn_2_O_3_ NFs have been also reported as efficient platforms for the immobilization of GOx or DNA probes in view of the electrochemical detection of glucose [38] or dengue consensus primer [71]. Mn salts and PVP [38] or PAN [71] were used as inorganic precursors and sacrificial polymers, respectively. In the first work, AgNO_3_ was also added to the electrospinning solution. The morphology of Mn_2_O_3_-Ag NFs produced after calcination of electrospun PVP/Mn(NO_3_)_2_/AgNO_3_ was different from that of the Mn_2_O_3_ NFs obtained from PVP/Mn(NO_3_)_2_. Ag NPs coalesced together, generating highly porous NFs with enhanced enzyme loading capacity and improved electrochemical features, could be observed by TEM. The NFs, dispersed into a 1 wt % Nafion solution and mixed with GOx, were further dropped onto the electrode and GOx was finally cross-linked using glutaraldehyde (GA) vapour. Glucose could be detected by amperometry with a low limit of detection (1.73 µM) and selectivity towards uric and ascorbic acids was demonstrated. In the second work, label-free zeptomolar detection of DNA hybridization was achieved and a dengue consensus primer was successfully quantified both in control and spiked serum samples (limit of detection: 120 × 10^−21^ M).

**Figure 3:**
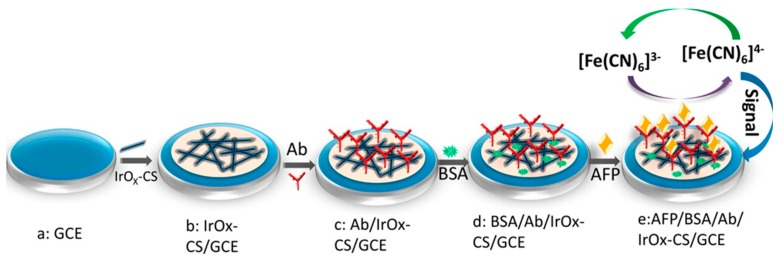
Principle of the electrospun NFs-based immunosensor for amperometric detection α-Fetoprotein proposed by Li et al. Reprinted with permission from [66]. Copyright 2015 American Chemical Society.

**Figure 4:**
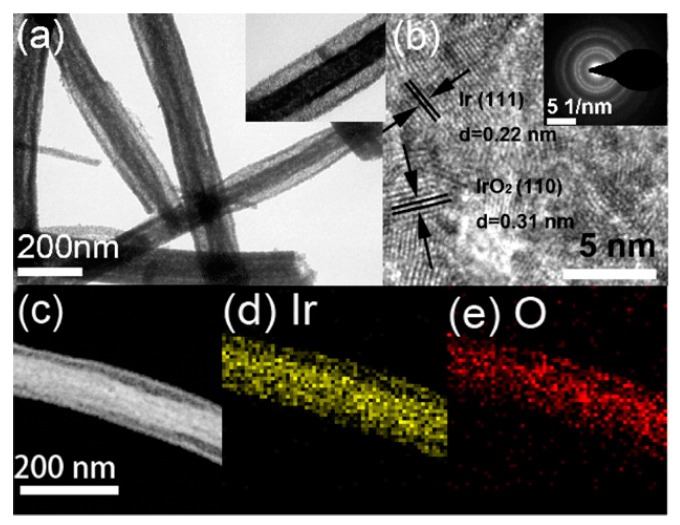
(**a**) TEM, (**b**) HRTEM, and (**c**) STEM images of IrO*x* nanofibers after annealing at 500 °C. The inset of panel (**a**) is the enlarged TEM image and inset of panel (**b**) is the SAED pattern. Panels (**d**,**e**) are the corresponding EDX mapping of Ir and O elements. Reprint with permission from [66]. Copyright 2015 American Chemical Society.

Li et al. [66] recently proposed a label-free immunosensor based on a glassy carbon electrode (GCE) modified by IrOx NFs/CS (Figure 3). IrOx composition (0 ≤ x ≤ 2) could be controlled by changing the annealing temperature. At 500 °C, wire-in-tube nanostructures of high surface area and rapid electron transfer kinetics were produced. TEM analysis evidenced that independent nanowires are embedded in the NFs and that the wire-intube nanostructures possess separated walls along nearly their entire length (Figure 4). The average diameter of the inside wire and whole NF was around 70 and 110 nm, respectively, and the detailed nanostructure can be observed more clearly in the inset of Figure 4a. Antibodies were adsorbed on the modified electrode and the biosensor was successfully used for amperometric detection of the cancer biomarker α-fetoprotein in human serum, with a limit of detection of 20 pg mL^−1^.

#### 3.1.2. Carbon NFs

Owing to its excellent mechanical and chemical resistance as well as its superior electronic properties, carbon is one of the most commonly used electrode materials in the elaboration of electrochemical sensors and biosensors. Carbon-based NMs, such as CNTs, have been extensively integrated within electrochemical biosensors to improve their sensitivity. Compared to CNTs, carbon electrospun NFs (CENFs) can provide higher surface areas for biomolecule immobilization and are more easily produced. They offer similar conductivities and can be modified to introduce suitable functional groups or combined with conducting NMs, e.g., metal NPs, to generate hydrophilic surfaces of enhanced electronic properties and larger biomolecules loading capacities.

Among the variety of polymers usable to produce CENFs, PAN is the most commonly employed, as it offers both a high carbon yield and the capacity to generate NFs of superior mechanical properties [39,40,41]. Carbonaceous NFs are fabricated from electrospun polymer NFs following a two-step process. First, NFs are stabilized at relatively low temperatures (200–300 °C) in an oxidative atmosphere to convert thermoplastic polymer NFs to condensed thermosetting NFs. Second, the NFs are carbonized at higher temperatures (typically 800–1300 °C) in an inert atmosphere. The microstructural and electronic properties of the fibers as well as their surface chemistry can be modulated by changing the composition of the electrospun solution, adapting the thermal treatment conditions or by adding a suitable post-modification process.

**Figure 5:**
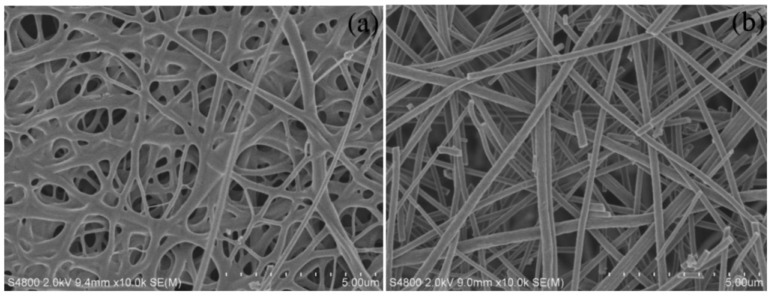
SEM images of (**a**) CENFs and (**b**) Cu/CENFs. Reprint with permission from [42].

Doping CENFs with metallic nanoparticles (MNPs), for example, has been shown to be an efficient method to enhance the sensitivity and stability of CENF-based biosensors. CENF-MNP composites are generally produced by electrospinning a precursor containing the polymer and a metal salt, the latter being converted into MNPs during the thermal treatment process [88]. Fu et al. [42] reported the fabrication of CENFs doped with Cu from a solution containing PAN, PVP and copper acetate. The electrospun NFs were further calcinated and dispersed in 0.1 M acetate buffer (pH 4), mixed with a laccase (Lac)/Nafion solution and casted on a glassy carbon electrode for amperometric detection of catechol. A CENF/Lac/Nafion/GCE without Cu was also produced for comparison. The CENFs, as observed by SEM (Figure 5a), exhibited a fibrous structure with an average diameter of 170 nm and fiber to fiber interconnections due to PVP carbonization.

In comparison, Cu NP/CNFs were more uniform, did not exhibit any interconnections, and the average diameter was significantly higher (300 nm, Figure 5b). The Cu NPs/CENFs/Lac/Nafion/GCE response to catechol was linear over a wide range of concentrations (9.95 µM–9.76 mM) and a low LOD (1.18 μM) was achieved, this value being 2.8 times lower than the LOD obtained in absence of Cu NPs. The stability was also slightly improved by loading the NFs with Cu NPs. Cu NPs/CENFs/Lac/Nafion biosensor retained 95.9% of the initial response after 22 days, while the CENFs/Lac/Nafion biosensor ended up with only 89.1%. The same group proposed another strategy to improve the performance of the biosensor [43]. The NFs were doped with Ni instead of Cu. A PAN-Ni acetate solution was first electrospun, then carbonized into a nitrogen atmosphere, and the resulting Ni NPs/ECNFs were mixed, after grinding, with Lac and dopamine (DA). The Lac-catalyzed oxidation of DA enabled the production of polydopamine (PDA) which efficiently embedded the enzymes into the Ni NPs/ECFs composite. PDA/Lac/Ni NPs/ECNFs aqueous suspension was finally casted onto a bare magnetic GCE and the resulting biosensor was assessed for catechol detection. Again, a control biosensor without Ni was prepared. LOD was improved by a factor of 1.7, compared to that of the Cu-doped CENFs mentioned before, and the linear range was also slightly wider (1 μM–9.1 mM). A good selectivity and stability was observed and the biosensor was successfully applied to the detection of catechol in spiked real water samples.

In another study, nitrogen-doped carbon nanosphere/ECNF composites (NCNS@ECNFs) were prepared by electrospinning polypyrrole nanosphere/PAN suspensions with subsequent controlled thermal treatment in a N_2_ atmosphere and GOx immobilization by absorption [44]. Then, the NCNSs@ECNFs composite was fixed on a GCE and recovered with Nafion. Doping CNSs with nitrogen was an effective way to improve their hydrophilicity (and therefore biocompatibility), as well as their electron-donor ability and electrical conductivity. Combining the large surface area of ECNFs and the enhanced electrocatalytic activity of NCNSs allowed the development of a sensitive, stable and selective glucose biosensor based on the direct electron transfer of GOx, with a low LOD (2 μM) and wide linear range (12–1000 μM). SEM images revealed that the NCNS@ECNFs exhibit a three-dimensional incompact porous structure, providing a large effective area for GOx immobilization. NCNSs of 53 ± 9 nm average diameter, well-dispersed on the surface of ECNFs or embedded within the matrix, could be also observed. The hydrophilicity of the ECNF surface was considerably increased by incorporating NCNSs, creating a favorable environment for GOx and for the direct electron transfer between the enzyme and the carbon electrode. The electron-transfer was higher with NCNS@CNFs/GCE than with the bare GCE or the CNFs/GCE, demonstrating the positive effect of doping.

Another method to produce ECNF-based enzyme biosensors with enhanced analytical performances was recently reported by Bae et al. [45]. Higher and controlled porosity was generated by incorporating silica NPs (average size: 16 ± 2 nm) into the precursor PAN solution. After electrospinning, the NPs were removed by HF treatment and the resulting PAN NFs were carbonized. Mesosized pore carbon structures fabricated this way were very beneficial for an efficient immobilization of GOx and the embedded enzyme remained very accessible to glucose substrate. Characterization of the ECNFs by Raman spectroscopy evidenced the positive effect of heat treatment on the crystallinity and orientation of carbon, and a significant increase of the conductivity was observed after thermal treatment.

Another strategy to improve bioreceptor loading or attachment to the ECNFs, and therefore biosensors’ analytical features, is to introduce post-treatments of the fibers using physical or wet chemical processes.

Cui et al. [46] functionalized carbonized PAN NFs with carboxylic groups using a wet chemical acidic treatment and used the carboxylated CENFs to synthetize hydroxyapatite (HA)-CENFs. Cytochrome *c*, further cast onto the microporous HA-CENFs composite, exhibited good electrocatalytic activity and fast response to H_2_O_2_.

Wang et al. [47] used the same protocol to produce ECNFs. Prussian Blue (PB) nanostructures could be subsequently grown in a controllable manner onto the carboxylic group-functionalized ECNFs. The PB-ECEF composite, coated on the surface of a GCE, was covered with a GOx/Chit film and an amperometric biosensor offering a wide linear range (0.02–12 mM) and low limit of detection (0.5 µM) was proposed for glucose detection.

Mondal et al. [67] first electrospun PMMA onto Si substrate, then the NFs were covered with a PAN/chloroplatinic acid film. High-temperature treatment of the composite film decomposed the PMMA NFs and generated embedded microchannels in the PAN-derived amorphous monolithic carbon electrode. The channels were decorated with Pt NPs by in situ thermal decomposition of the precursor metal salt. Carboxylic groups were generated at the composite surface by plasma treatment and anti-aflatoxinB1 (AFB1) antibodies were grafted using the EDC/NHS conjugation chemistry. The nanochannels aligned in the porous carbon film acted as a reaction chamber for antigen–antibody interactions and was beneficial for fast electron transport toward the electrode. AFB1 could be detected as low as 1 pg/mL with a linear range of 10^−12^–10^−7^ g/mL.

Kim et al. [74] fabricated a potentiometric aptasensor based on ECNFs for bisphenol A (BPA) detection. Electrospinning of a PAN/PPMA phase-separated blend, followed by thermal treatment, generated multi-channel ECNFs of large surface area, clearly evidenced by SEM. The fibers were subsequently functionalized with carboxylic groups using an acidic oxidative treatment and BPA-binding aptamers were attached to the surface via covalent binding. The biosensor was highly sensitive (LOD: 1 fM) and could be reused over a period of 4 weeks.

#### 3.1.3. Electrospun Polymeric and Composite NFs

As already mentioned, among the NF-based biosensors reported in the literature, only a few of them rely on optical [61,69] or mechanical [70] detection modes, and most of them are associated with electrochemical transduction. In this case, NFs must be conductive. Apart from the metal-oxide and carbon NFs described in the previous sections, composite NFs based on polymers incorporating conductive materials have been reported. The conductive materials may be metallic NPs (MNPs), carbon nanotubes (CNTs), conducting polymers or combinations [28].

##### Electrospun Polymer NFs Doped with CNTs

The straightforward strategy to produce CNT-polymer NF composites is to disperse CNTs into the polymer solution before electrospinning. Following this approach, Manesh et al. [48] fabricated a glucose biosensor by immobilizing GOx onto a polymer-CNTs composite. Multiwall carbon nanotubes (MWCNTs), wrapped by a cationic polymer [poly(diallyldimethylammonium chloride)] (PDDA), were dispersed into PMMA, and a nanofibrous membrane was produced by electrospinning the blend onto an ITO electrode. Wrapping PDDA over the surface of the MWCNTs helped prevent the aggregation of the MWCNTs and was used to attach the negatively charged enzyme onto the modified electrode surface. A thin layer of Nafion was added over the electrode surface to decrease the interferences caused by anions present in biological media. The fabricated Nafion/GOx/MWCNT(PDDA)-PMMA NFs/ITO electrode exhibited excellent electrocatalytic activity towards hydrogen peroxide (H_2_O_2_) with a pronounced oxidation current at +100 mV. Glucose was amperometrically detected at +100 mV in 0.1 M phosphate buffer solution (PBS, pH 7), with a fast response time (4 s). The response to glucose was linear in the 20 µM–15 mM range and LOD was 1 µM.

Wang et al. [49] prepared CNT-doped poly(acrylonitrile-co-acrylic acid) (PANCAA) NFs by electrospinning and further modified the NFs with GOx by covalently immobilizing GOx on the membranes through the activation of carboxylic groups on the PANCAA NF surface. The electrochemical properties of enzyme electrodes were characterized by chronoamperometric measurements, which showed that MWCNT filling enhances the electrode current and sensitivity. Combined with the results of kinetic studies, it was concluded that the interactions between MWCNT and FAD play a significant role in enhancing the electroactivity of the immobilized GOx, even though the secondary structure of the immobilized GOx is disturbed in the presence of MWCNT.

Numnuan et al. [50] proposed an amperometric biosensor based on electrospun Chit-CNTs NFs for uric acid detection. A Ag NPs layer was first electrodeposited on a gold electrode. A Chit/PVA/MWCNTs mixture was then electrospun and, after removal of PVA by NaOH treatment, uricase was immobilized on the Chit-CNTs NF film through cross-linking between its amine groups and the Chit amino groups. The fabricated uric acid biosensor had a wide linear range (1.0–400 µM), with a LOD of 1.0 µM and a storage life of more than six weeks. The values measured for blood plasma samples using the proposed biosensor were in good agreement with those obtained by a standard enzymatic colorimetric method.

More recently, Bourourou et al. [51] dispersed MWCNTs into a PAN solution to produce electrospun NF mats that were used directly as electrodes. The nitrile groups of PAN polymer were subsequently reduced into amino groups, and polyphenol oxidase (PPO) was immobilized onto the PAN-MWCNTs NFs through covalent bindings using GA. The PAN-MWCNTs-PPO electrode was successfully used for the sensitive amperometric detection of catechol, with a wide linear range (1 µM–0.4 mM) and a LOD of 0.9 µM.

##### Electrospun Polymer NFs Doped with MNPs

The electrochemical properties of MNPs are extremely sensitive to their sizes, shape, and dispersion. A high dispersion of MNPs in functional materials is important to provide high electrochemical activity, while the associated tendency of MNPs to aggregate would lower their catalytic activity and reuse lifetime. Therefore, how to design and prepare MNP-based materials with long-term dispersion stability and high catalytic efficiency is a primary challenge for their wide applications. MNP-doped NFs can be prepared, as described for CNTs, by dispersing the NPs into a polymer solution and spinning subsequently. Devadoss et al. [52] synthetized Au NPs-Nafion-polyacrylic acid (PAA) NFs by electrospinning a blend of the three components. *N*,*N*′(4-dimethylamino) pyridine (DMAP)-protected Au NPs (5.0 ± 0.5 nm) were used. TEM images evidenced the uniform inclusion of Au NPs in the composite NFs, which was attributed to a strong electrostatic interaction between the positively charged DMAP-protected Au NPs and the negatively charged sulfonate groups in Nafion. Au NP-composite NFs exhibited higher conductivity than the Nafion-PAA NFs. Horseradish peroxidase (HRP) was further immobilized on the nanofibrous electrode via electrostatic interactions with the negatively charges of PAA. It was demonstrated that the incorporation of Au NPs in the NFs improved the amperometric detection of H_2_O_2_. The biosensor LOD was decreased by a factor of 2.6.

**Figure 6:**
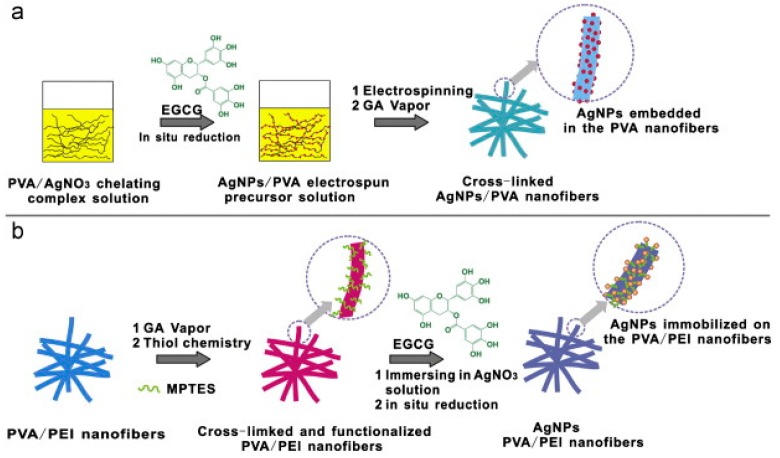
Fabrication process of the (**a**) AgNPs embedded in the PVA water-stable nanofibers and (**b**) AgNPs immobilized on the functionalized PVA/PEI water-stable nanofibers Reprint with permission from [53]. Copyright 2013 Elsevier.

MNPs-polymer hybrid NFs can be also synthetized through in situ reduction of metallic precursor ions, either introduced in the electrospun solution, or dropped on the polymer NFs after electrospinning. Zhu et al. [53] reported a facile and green approach to prepare Ag NPs-PVA NFs and Ag NPs-PVA/PEI NFs using the first and second strategy, respectively (Figure 6). The freshly prepared NFs were cross-linked using GA vapors to improve their water stability. Ag NPs were generated within the PVA NFs or on the surface of the PVA/PEI NFs by in situ reduction of AgNO_3_ precursor with the green reductant, epigallocatechin gallate. Then, HRP was dropped onto the fiber mats. The HRP/AgNPs/PVA/GCE and HRP/AgNPs/(PVA/PEI)/GCE biosensors exhibited high amperometric sensitivity to H_2_O_2_ and glucose, the best analytical performances being obtained for the HRP/AgNPs/(PVA/PEI)/GCE biosensor. In another work of the group, PVA/PEI NFs were elaborated and immersed into a PdCl_2_ solution for subsequent reduction of the Pd salt with NaBH_4_ [54]. TEM characterization evidenced a good dispersion of Pd NPs on the NFs, which was attributed to the complexation between Pd(II) and the free amine groups of PEI. The average diameter of the produced Pd NPs was 3.4 nm, some aggregation being noticed. The HRP/AgNPs/PVA/PEI NFs/GCE biosensor, obtained after adsorption of HRP, exhibited higher CV response to H_2_O_2_ than the HRP/AgNPs/PVA/PEI NFs/GCE biosensor, demonstrating that Pd NPs play a key and positive role in the electron transfer between the redox-active site of H_2_O_2_ and the electrode. Jose et al. [55] proposed to combine the advantages of electrospinning with those of both CNTs and Au NPs to produce an amperometric glucose biosensor of enhanced performances. In this approach, PAN NFs_,_ were first decorated with Au NPs using a seed-mediated electroless deposition method. Carboxylated MWCNTs were further coated onto the Ag NPs/PAN NFs by electrophoretic deposition. SEM images revealed a complete and uniform coating of the fibers surface with MWCNTs. The carboxylated MWCNTs provided the anchor for covalent immobilization of GOX. The direct electron transfer between GOx and the electrode surface was demonstrated. The LOD for glucose was as low as 4 µM.

##### Conducting Polymers in the Fabrication of Electrospun NFs

Conducting polymers (CPs), such as polyaniline (PANI), polypyrrole (PPy) or poly(3,4-ethylenedioxythiophene) (PEDOT) are of particular interest in the elaboration of electrochemical biosensors due to their unique electrical properties. They possess fascinating chemical and physical properties, such as intrinsic conductivity, derived from their conjugated π-electron system and so they have been used to enhance the speed, sensitivity and versatility of many biosensors. Direct electrospinning of CPs would therefore be a facile and rapid way to create conducting NFs. However, processing intrinsically conducting polymers has always represented a challenge. Indeed, most of them are insoluble and infusible due to the stiffness of their all-conjugated aromatic backbone structures, which renders them hardly electrospinnable.

To overcome this issue, different approaches may be used. The first strategy consists in blending the CP with a non-conducting spinnable polymer. The latter serves as a carrier to improve the CP spinnability. Gladish et al. [56] created polymer NFs based platforms for enzymes immobilization by electrospinning blends of PAN and highly conductive sulfonated PANIs on ITO electrodes. Apart from sulfonic acid groups, the polymers exhibit carboxylic acid groups which were used for covalent immobilization of the pyrroloquinoline quinone-dependent glucose dehydrogenase (PQQ-GDH). The modified electrodes demonstrated high catalytic current responses to glucose and a wide range of detection (2.5 µM–1 mM) could be achieved.

**Figure 7:**
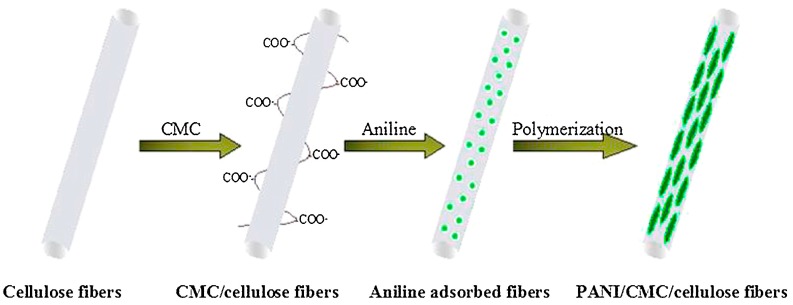
Preparation of PANI/CMC/cellulose nanofibers. Reprinted with permission from [57]. Copyright 2015 Elsevier.

**Figure 8:**
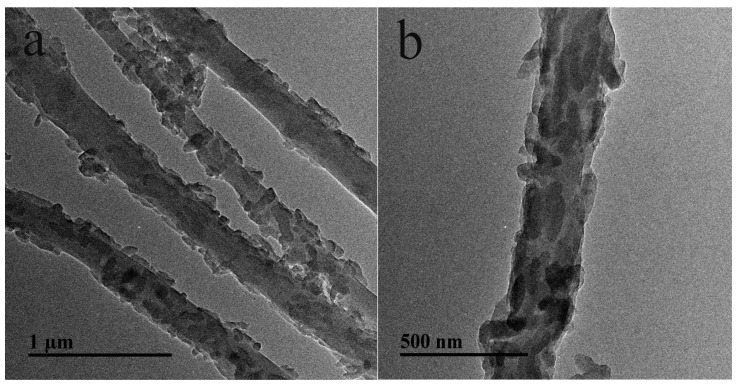
TEM images of PANI/CMC/cellulose nanofibers at different magnifications. Reprinted with permission from [57]. Copyright 2015 Elsevier.

In a second approach, electrospun NFs are covered with a CP. Fu et al. [57] prepared hierarchical PANI/carboxymethyl cellulose (CMC)/cellulose NFs on GCE electrodes by in situ polymerization of aniline on the CMC-modified cellulose NFs (Figure 7). Highly dense PANI nanorods (60 nm × 180 nm) grown onto the surface of CMC/cellulose NFs could be visualized by TEM (Figure 8). The NFs exhibited an average diameter of 310 ± 8 nm. A Nafion/Lac solution was dropped at the NFs surface for Lac fixation. The fabricated Lac/PANI/CMC/cellulose/GCE exhibited a highly sensitive detection toward catechol with a broad linear range and low detection limit (0.374 µM).

In a similar approach, electrospun poly(L-lactide) (PLLA) NFs were produced onto Pt microelectrode arrays and covered with GOx-PEDOT films by electropolymerization of EDOT monomer in presence of poly(sodium-*p*-styrene sulfonate) and GOx [58]. The analytical performances of the NFs modified electrodes were compared to those of Pt electrodes covered with PEDOT/GOx films of similar thickness (around 330 nm). In addition to improved sensitivity, the PEDOT NFs-GOx biosensors exhibited a lower LOD for amperometric glucose detection (0.12 mM at +700 mV) than the PEDOT film-GOx biosensors (0.45 mM). Guler et al. [72] also reported the fabrication of poly(ε-caprolactone) (PCL) NFs onto an ITO-PET electrode followed by in situ chemical polymerization of pyrrole with Fe^3+^ as an oxidant and Cl^−^ as a dopant. The PCL/PPy NFs were functionalized with calf thymus ssDNA, used as model, by physical immobilization. Different techniques evidenced the effective incorporation of PPy and ssDNA on PCL NFs, and demonstrated the possibility of using the PCL/PPy NFs as electrochemical DNA biosensor after immobilization of a specific probe DNA onto the fibers.

To improve the electrochemical properties of the final nanofibrous membranes, some authors proposed to make the electrospun polymer conductive by integrating CNTs into the electrospun solution. Uzun et al. [59] fabricated a glucose sensor by preparing electrospun nylon 6,6 NFs incorporating MWCNTs, which were further coated with a conducting polymer named PBIBA [poly-4-(4,7-di(thiophen-2-yl)-1*H*-benzo[d]imidazol-2-yl)benzaldehyde] to covalently attach GOx on the surface of the fibers through the free aldehyde groups of the conducting polymer. The resulting novel amperometric glucose biosensor revealed higher stability and sensitivity in presence of MWCNTs. The linear response for glucose detection is in the range of 0.01 mM to 2 mM with a LOD of 9 μM. In the same way, MWCNTs-doped nylon 6,6 composite NFs were prepared using electrospinning, and served as backbone for pyrrole electropolymerization [73]. The functional composite was used to immobilize wild type p53 ssDNA for hybridization detection of a specific mutation in the p53 tumor suppressor gene. Ekabutr et al. [60] reported the fabrication of PAN-MWCNTs hybrid NFs on a screen-printed carbon electrode. The NFs were covered with PPy using an original approach by vapour phase polymerization (VPP) of pyrrole using Fe(III) *p*-toluenesulfonate as oxidizing agent. GOx, used as a model enzyme, was subsequently adsorbed on the modified electrode and the biosensor was evaluated for amperometric detection of glucose. The LOD was 0.98 mM. The VPP approach was also employed in another study by Jun et al. [75] to cover carbon NFs scaffold decorated with ZnO nanonodules with carboxylated PPy. The platelet-derived growth factor-B (PDGF-B) binding aptamer was conjugated to the NFs at the surface of a FET transducer via PPy carboxyle groups. The biosensor was highly sensitive (5 fM) and extremely selective for isoforms of PDGFs.

### 3.2. Enzyme Entrapment into the NFs

When enzymes are attached to NFs after electrospinning, only the external surface of the NFs may be used for immobilization, without taking full advantage of the internal volume of fibers that can protect enzyme molecules from harsh conditions. Enzyme loading is therefore rather limited. Therefore, another way to proceed consists in electrospinning a blend of enzymes and water-soluble polymer. This approach might be also used to entrap antibodies, but has been rarely investigated due to the high amount of biomolecules required and their high cost [31]. In contrast to the protocols described in the previous sections, the entrapment does not involve any bond between the enzyme and the NFs surface that helps preserving their biological activity [77]. The major drawback of the approach is that some enzymes may be shielded inside the NFs and are not accessible to the substrate. A summary of the enzymatic biosensors prepared this way may be found in Table 1.

PVA is the most common water-soluble polymer employed for enzyme entrapment into NFs as it is a non-toxic, hydrophilic and highly biocompatible synthetic polymer. However, data reported in the literature are not easy to compare. Indeed, PVA displays various degrees of polymerization and hydrolysis (DP and DH, respectively). These parameters highly affect the viscosity and conductivity of PVA solutions, and therefore the electrospinning conditions and the morphology of electrospun NFs produced. Ren et al. [78] proposed an amperometric glucose biosensor prepared by electrospinning a mixture of PVA and GOx in PBS followed by cross-linking using GA in solution to form water-insoluble NFs. The NF diameters obtained in this work were somewhat heterogeneous, ranging from 70 nm to 250 nm in the absence of GOx, as observed by SEM. In the presence of GOx, the fibers became irregular and were interspersed with shuttle-shape beads. The presence of GOx in the NFs was evidenced by IR spectroscopy. The amperometric response to 0.5 mM glucose was more rapid (1 s) than the response observed for PVA/GOx films (5.2 s) under the same conditions. The enzymes remained active when entrapped in the NFs and a LOD of 0.05 mM was measured. Oriero et al. [79] immobilized tyrosinase onto an ITO-coated glass substrate modified with silica-tyrosinase-PVA NFs for amperometric detection of catechol, phenol, and *p*-cresol. The biosensor sensitivity decreased in the order catechol > phenol > *p*-cresol, with a LOD of about 10 μM for each compound and a linear response up to 100 mM.

To enhance the conductivity of PVA NFs, the incorporation of carbon NMs, mostly graphene oxide (GO) and MWCNTs, or Au NPs has been proposed. Su et al. [80] developed a novel biosensing platform for glucose detection, by electrospinning a solution containing PVA, Chit, GO and GOx as model enzyme to fabricate NFs. After electrospinning, the Pt electrode modified with electrospun Chit-GOx-GO-PVA NFs was placed in glutaraldehyde vapor for cross-linking to form water-insoluble NFs. Then, a thin layer of Nafion was deposited on the surface of the matrix, and the prepared electrode was used for glucose amperometric detection. The electrode exhibited high sensitivity, good stability, low detection limit (5 μM) and wide linear range (5 μM–4 mM). Glucose concentrations determined in human serum samples by the NF-based biosensor were in good agreement with the values obtained using a standard clinical assay. The same methodology was used to entrap choline oxidase. The choline biosensor exhibited excellent analytical features, demonstrating the versatility of the proposed method. A GOx-GO-PVA NFs modified Pt electrode was also constructed for glucose detection [81]. The highest sensitivity was obtained by incorporating 20 ppm GO in the electrospun solution. The biosensor response was very rapid, the steady-state current being achieved only 10–14 s after glucose injection.

**Figure 9:**
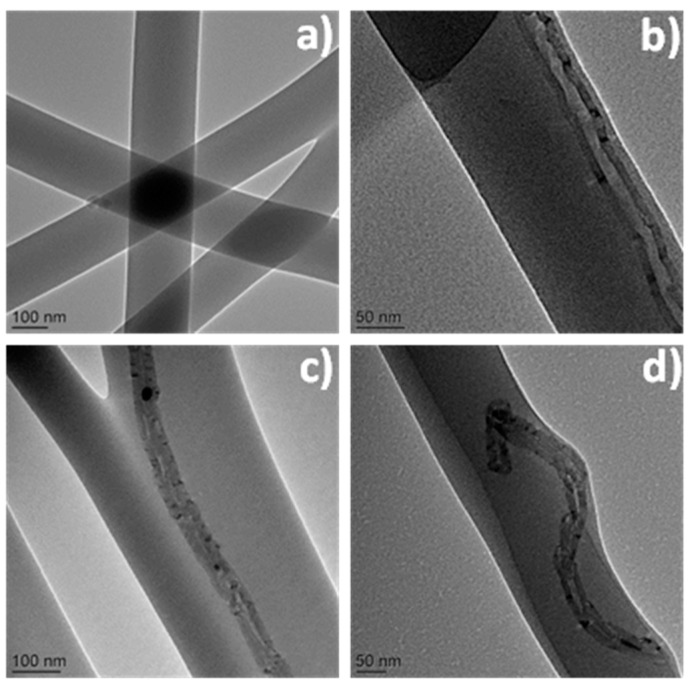
TEM images of (**a**) pure PVA-SbQ NFs, (**b**) PVA-SbQ/MWCNTs (1 wt %) NFs, (**c**) PVA-SbQ/MWCNTs (5 wt %) NFs, (**d**) PVA-SbQ/MWCNTs (10 wt %). Reprinted with permission from [82]. Copyright 2015 The Electrochemical Society

Our group has proposed two strategies to produce enzyme NF-based electrochemical biosensors from enzymes-polymer blends [82]. GOx was chosen as model enzyme. In a first approach, we reported the one-step and facile fabrication of water-stable NFs from blends of the photochemically cross-linkable polyvinyl alcohol styrylpyridinium polymer (PVA-SbQ), MWCNT and GOx. MWCNTs were functionalized with carboxyl groups to improve their compatibility with PVA and PVA/GOx aqueous solutions The NFs were stabilized by UV-cross-linking of PVA-SbQ, which enabled a fast, easy and soft cross-linking step without any added chemicals. The enzyme conformation and activity could be preserved. The addition of MWCNT resulted in a significant decrease in the average fiber diameter, from 350 ± 20 nm to 250 ± 50 nm, as evidenced by SEM. In addition, embedded nanotubes, well dispersed in the polymer matrix and aligned along the NFs direction, could be detected by TEM for the lowest MWCNT loadings (Figure 9b,c). MWCNTs tended to agglomerate at the highest MWCNTs loading, and clusters of coiled/bundles nanotubes were observed (Figure 9d). The combination of MWCNT and PVA-SbQ polymer improved electron transfer ability of the generated nanofibrous mats. The resulting enzyme voltammetric biosensor was linear in a wide range of glucose concentration (up to at least 4 mM) and a very low LOD (2 µM) was achieved.

**Figure 10:**
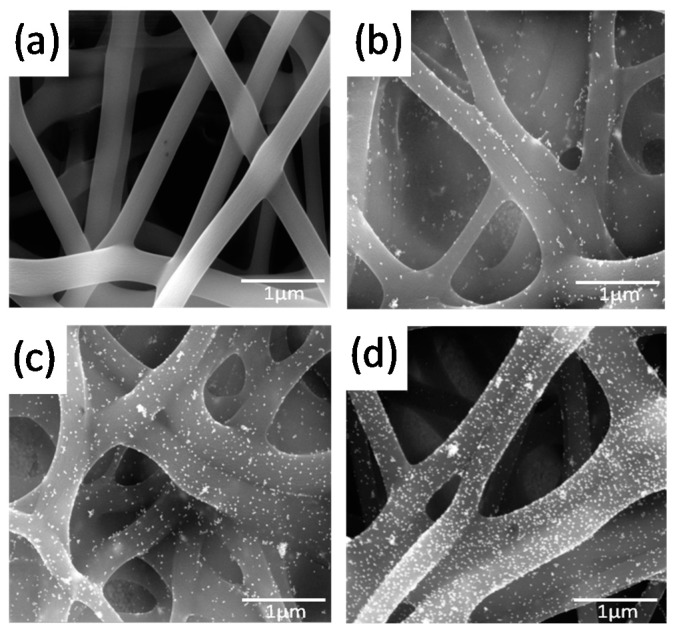
SEM images of water-stable electrospun PVA/PEI NFs before immersion in the Au NPs solution (**a**), after immersion in colloidal Au NPs solutions of pH 7.0 (**b**), pH 6.0 (**c**), pH 5.0 (**d**). Reprinted with permission from [83]. Copyright 2016 Elsevier.

In a second study [83], aqueous solutions of GOx-PEI-PVA were electrospun on Au electrodes and the NFs were decorated with Au NPs. It was shown that adhesion of the fibers mat onto gold electrodes could be improved by modifying the surface with a self-assembled monolayer of 4-ATP bearing thiol groups for covalent binding to the gold surface and amine groups to react with the amine groups of PEI in a subsequent cross-linking step. This step also helped improving the water stability of the produced NFs. A significant enhancement of the NFs’ conductive properties, as characterized by cyclic voltammetry, was achieved by decorating the NFs with AuNPs. The highest density of particles was observed using Au colloidal solutions of pH 5, which could be attributed to hydrogen bondings and ionic interactions between the amine groups of PEI and the carboxylic groups of citrate-stabilized Au NPs (Figure 10). Glucose could be successfully detected by impedimetry, the response being linear in the 10–200 µM range. A very low limit of detection (0.9 µM) could be achieved and the biosensor exhibited good operational and storage stabilities.

## 4. Electrospun NFs in ELISA-Type Bioassays and Point-of-Care (POC) Devices

Owing to their high surface area and their ability to bind biomolecules, electrospun NFs have also been exploited to the elaboration of improved Enzyme-Linked ImmunoSorbent Assays (ELISAs) and of advanced point-of-care (POC) devices. The analytical performances of these devices of course depends strongly on the immobilization of the affinity elements (mostly antibodies) to the assay substrate. Random adsorption is by far the most common immobilization technique, but bioaffinity attachment, using for example the specific binding of biotin to avidin and streptavidin, is also relatively widespread [89]. Covalent attachment has been also investigated to limit antibodies leaching and loss of functionality that physical adsorption could induce, and to achieve a better distribution of the biomolecules onto the surface. Both electrochemical and optical detection are employed, with a predominance of the latter.

ELISA is perhaps the most well-known and widely used immunoassay for the detection of proteins (antibodies, biomarker proteins), pathogens (bacteria, virus) and smaller molecules of toxicological or environmental interest. However, it suffers from several major weaknesses. The conventional test is time-consuming and laborious, requires large amounts of sample and exhibits low sensitivity, which impedes early diagnosis of diseases. In addition, it is a single target analyte test, whereas it is known, for example, that the combined measurement of several biomarkers is required to increase the accuracy of disease diagnosis. Finally, samples must be purified before the test in order to avoid interferences. Different strategies have been proposed to overcome these different issues, resulting in the development of improved ELISA-type assays and advanced POC devices, among which lab-on-a-chip/lab-on-a-disc devices, lateral flow and microfluidic paper-based bioassays [90,91,92,93,94]. The following sections will show how the integration of electrospun NFs in such systems can contribute to improve their performances.

### 4.1. ELISA-Type Bioassays

Conventional ELISA relies on two-dimensional surfaces of planar substrates to immobilize the capture molecules. Due to their large surface area-to-volume ratio, the three-dimensional electrospun NF mats have the capacity to immobilize larger amounts of capture molecules, resulting in higher expected sensitivities. Wang et al. [95] recently proposed a high-throughput immunoassay based on electrospun PS NFs and compared its performance with conventional PS substrate. A plasma treatment was applied to make PS NFs hydrophilic, facilitating infiltration of antibodies into the NFs scaffold. Three different cancer biomarkers (AFP, CEA, VEGF) were further used to compare the analytical performance of the two substrates using a sandwich approach and fluorescence detection. A 300-fold decrease of the LOD was achieved by replacing the planar PS substrate by electrospun PS NFs.

Other polymers of various levels of hydrophilicity and functionalities have been investigated to evaluate their ability to generate sensitive and more rapid ELISA. Sadir et al. [96] produced NF mats from two polymers: poly-l-lactic acid (PLLA) and cellulose acetate (CA). The CA NFs were treated with GA to enhance their water stability. Electrospun NF membranes were placed in a microwell plate and the sensitivity of the two bioassay platforms were compared for the detection of CRP cardiac biomarker using a colorimetric sandwich ELISA approach. The LODs were 13 pg mL^−1^ and 53 pg mL^−1^ for PLLA and CA, respectively, lower than that of the conventional ELISA. Moreover, the total analysis time was reduced by a factor 2. It was shown that the amount of antibodies adsorbed on PLLA was higher than on CA and that the proteins were more stable. In another work, polyhydroxybutyrate (PHB) NF membranes were produced by electrospinning and were coated with poly(methylmethacrylate-co-methacrylic acid) co-polymer, poly(MMA-coMAA) [97], which was synthetized with different molar ratios of the monomers, to achieve nanofiber membranes combining the high surface area of PHB NFs with functional carboxylic groups inherited from MMA segments of the copolymer. The hydrophobicity of PHB NFs gradually decreased and the number of functional groups increased as the number of MAA segments introduced via poly(MMA-co-MAA) coating increased. The coated and uncoated PHB NF membranes were placed into a PS ELISA well-plate for subsequent colorimetric detection of dengue virus using a sandwich approach. Two modes of primary antibodies immobilization were tested, i.e., physical adsorption and covalent binding through EDC/NHS chemistry method. Covalent binding generally yielded lower signals than physical adorption but higher than conventional ELISA. The coated NFs with 9:1 MMA:MAA molar ratio of exhibited the lowest LOD and highest specificity. In a recent work, Mahmoudifard et al. [98] reported the fabrication of electrospun NF mats from the hydrophobic polysulfone polymer. Oxygen plasma treatment enabled the creation of carboxylic functional groups at the surface of the nanofibrous membrane and monoclonal IgG antibodies were further immobilized covalently or by physical adsorption to perform a simple colorimetric sandwich ELISA in well-plate format. Covalent attachment resulted in higher signals compared to those obtained with adsorption approach. In addition, it was shown that the proposed platform integrating electrospun NFs was more sensitive than the standard ELISA.

Polymers with biocompatible and antifouling properties have been also proposed to reduce the background noise coming from nonspecific adsorption of proteins in ELISA. Using electrospun NFs prepared from a functional polymer bearing both phosphorylcholine and active ester groups, Chantasiricot et al. [99] developed an ELISA for human IgG detection. The LOD was improved by a factor 4.6 compared to the conventional ELISA performed on PS substrate and 1.8 compared to the test performed with a dip-coated polymer substrate. A much wider linear range could be also achieved. In addition, the blocking step could be omitted, resulting in a significant decrease of the total time of analysis. Hersey et al. [100] synthetized a set of water insoluble poly(oxanorbornene) derivatives with biotin (bioactive) and triethylene glycol (antifouling) functionalities. Electrospun NF meshes were generated from these copolymers with the ability to specifically bind streptavidin while minimizing the nonspecific binding of other proteins. The protein binding capabilities of NF meshes were evaluated using a simplified colorimetric ELISA for a model target protein, mouse IgG, under both static (26 h) and flow conditions (1 h). Under flow conditions, the LOD was 2.2-fold lower than the LOD of the conventional ELISA in streptavidin coated plates.

Recent works have also been devoted to the development of original microarray-based immunoassays using either hydrogel-micropatterned PS-based NFs [101] or droplets immobilized on polycaprolactone NFs [102].

### 4.2. Lab-on-a Chip/Lab-on-a-Disc POC Devices

The integration of bioaffinity tests into more compact, rapid, simple and easy-to-use devices usable on-site by non-qualified persons have been the subject of tremendous research works over the past decades, resulting in the development of POC devices. In these devices, minimal human intervention should be achieved, ideally only for sample introduction, signal read-out and interpretation. The consumption of minimal volumes of sample is also targeted. Lab-on-a chip [90] or lab-on-a-disc [91] devices integrate sample preparation and detection in one system. They can address the requirement of providing a fast and multiplexed diagnostic at the point-of-need.

Electrospun NFs can contribute to the improvement of biosensing lab-on-chip devices in several ways. It has been shown recently that they can be very useful as passive mixers, with capacities comparable to or better than many passive mixers already reported [26]. Jin et al. [103] developed a capillary flow microfluidic bio-chip for chemiluminescence detection of *E. coli 0157* cells combining lateral flow assay and microfluidic technology, demonstrating the applicability of water soluble NFs for on-chip reagent delivery. In this work, PVP NFs containing HRP tagged antibodies were produced and incorporated into the chip and the delivery of binding reagents, i.e., antibodies, was achieved by dissolution of PVP NFs. Electrospun NFs can be also used to specifically capture and concentrate the analytes for enhanced purification and detection. One major difficulty comes from the integration of NFs into the microchannel of the lab-on-a-chip system. Cho et al. [104] fabricated patterned aligned PVA NFs using gold microelectrodes array into PMMA microchannels. The same group later evaluated the approach for specific liposomes retention [24]. Very recently, they proposed a more simple fabrication method, where thin layer of NFs were first prepared, then manually peeled off the grounded collector and stacked together to create multilayer mats with more homogeneous morphologies [25]. The mats were finally transferred to PMMA substrates, cut into small strips and incorporated into the microchannels. Positively and negatively charged PVA NFs were prepared. The negatively charged PVA mats bearing carboxylate groups was used to attach anti-*E. coli* K12 antibodies via EDC/NHS chemistry. Positively charged NFs enabled 87% retention of negative *E. coli* K12 bacterial cells through non-specific electrostatic interactions, while antibody-functionalized negatively mats were capable of the specific capture of 72% of the cells while reducing non-specific analyte retention due to the repealing effect of the polymer. This work therefore demonstrated the potential of the method for efficient capture and concentration of microbial cells. Lee et al. [105] exploited these attractive properties to immobilize high amounts of suitable antibodies on TiO_2_ NFs integrated in a lab-on-a-disc device. The TiO_2_ NFs had been previously treated with O_2_ plasma and functionalized for antibodies binding. The chemiluminescence biosensing device enabled the sensitive and rapid (30 min) detection of two cardiac biomarkers (CRP and cTnl). LODs were 0.8 pg/mL and 37 pg/mL, respectively, and the method required only 10 µL of sample. Ali et al. [106] reported the development of a label-free electrochemical immuno-biochip detection of breast cancer biomarkers. The biosensor utilized a uniquely structured working electrode made of porous hierarchical graphene foam modified with electrospun carbon-doped TiO_2_ NFs inserted in the microchannel of the PDMS microfluidic device. Antibodies were immobilized covalently onto the electrode. High sensitivity and specificity could be achieved.

### 4.3. Paper-Based POC Devices

Due to their low-cost and various easy-to-use formats, paper-based devices have appeared for many years as very appealing and cheaper alternatives to lab-on-a-chip systems. The porous structure of paper allows fluid flow by capillary action without the need of additional pumping systems. The paper-based devices exist under various formats, from the basic dipsticks and lateral flow assays (LFAs) to the more advanced and complex forms, i.e., microfluidic paper-based analytical devices, with possible multiplexing [92,93,94].

LFA format typically consist of four segments, including the sample pad, the conjugate pad, reaction membrane and an adsorbent, all being enclosed in a plastic cassette [93]. Luo et al. [107] reported the fabrication of a LFA using nitrocellulose nanofibrous membranes functionalized with antibodies directed against bovine viral diarrhea virus (BVDV) as the capture pad. The capillary action of the membrane was enhanced using oxygen plasma treatment. LFA was very rapid (8 min) and a good LOD (10^3^ CCID/mL) was obtained. Reinholt et al. [108] also demonstrated that PLA-based electrospun nanofibers could be successfully incorporated as platform in paper-based biological LFAs. Antibodies were attached to the NFs by adsorption and a colorimetric enzymatic sandwich immunoassay was developed for the detection of *E. coli* O157:H7.

## 5. Conclusions and Future Prospects

A number of electrospun NFs-based biosensors have been developed, using a large variety of approaches, polymers, integrating or not organic or inorganic fillers (e.g., conducting NMs) and post-treatment processes to generate robust biosensing platforms with enhanced functionalities and analytical performances. There is no doubt that the design and fabrication of NFs-based biosensors is an expanding area of research but it is still in an early stage of academic study in laboratory.

At present, most of the biosensors reported use enzymes as sensing elements and electrochemical transduction. Electrospun NFs are excellent supports for enzyme immobilization, providing large surface areas, porosity, functionalized surfaces for attachment or entrapment, favorable environments around the biomolecules to improve enzyme stability and activity. In addition, most enzymatic biosensors based on electrospun NFs have been designed and evaluated using a model enzyme, GOx. The high versatility of electrospinning process and of the methodologies developed open the way to their application to the immobilization of a wider range of biomolecules, i.e., other enzymes, antibodies, DNA or aptamers, and other transduction modes, e.g., optical. Table 2 shows that very good analytical performances, at least in terms of linear range and LOD, can be achieved for electrochemical detection of glucose using NF-modified electrodes. However, only a very limited number of them has been applied to real samples analysis and no commercial devices are yet available. Electrospun NFs have also demonstrated high potential for the elaboration of advanced POC devices, acting as efficient supports for antibodies capture, analyte concentration, signal amplification, with interesting capillary properties for integrating in paper-based devices.

Although significant progress has been made in the past decade in the field, there is still some areas where further improvements and refinements are required, i.e., a better control of the NF production, diameter and arrangement on the transducers, as well as of the immobilization of the biomolecules and of the physico-chemical and biological processes occurring at the NF-based biointerfaces. More fundamental and modeling approaches will be very helpful to better understand and control the interactions between the polymers, fillers and biomolecules (both before and after electrospinning), and the effect of the various electrospinning/immobilization parameters on the size, morphology and electrochemical/optical properties of the NF-based biohybrid materials produced. It will be also interesting to explore new strategies of producing NFs with enhanced electronic/optical properties or functionalities. An original approach has been recently proposed, which combines electrospinning and atomic layer deposition technique to generate controlled ZnO nanocristalline layers and ZnO/Al_2_O_3_ nanolaminates at the surface of PAN electrospun NFs used as template. The enhanced photolelectronic and photoluminescence features of the NFs allows applications in optical and electrochemical biosensors [109,110]. Gonzales et al. [111] reported the successful one-step production of a biotin surface functionalized hydrophilic non-water soluble PLA NFs. Incorporation of PLA-*b*-PEG copolymer together with biotin in the spinning solution significantly increased the amount of biotin available at the fibers surface able to bind avidin. The functional hydrophilic NFs proposed in this work are really promising for use in POC devices.

Finally, the conventional electrospinning process, used in most of the works reported, generates NFs of random orientation, limiting the repeatability of biosensors fabrication and therefore hampering a future reliable and consistent production at the industrial scale. Some strategies and advanced electrospinning set-ups have been recently proposed for a more accurate deposition of various patterns of polymer NFs with controlled orientation and spacing [10,84]. The development of new devices, such as coaxial set-ups, which enable the production of core-shell NFs, is also very promising in the biosensor area. Using this approach, Ji et al. [85] recently reported the fabrication of a colorimetric ready-to-use glucose test strip based on GOx and HRP co-immobilized with two commonly used chromogenic agents, in the hollow chamber or shell of polyurethane hollow NFs.

## Figures and Tables

**Table 1:** Electrospun NFs-based enzyme biosensors fabricated by attachment of the enzymes onto the fibers. Unless otherwise stated, the detection is performed by amperometry.

NFs	Treatment after Electrospinning	Bioreceptor (Analyte)	Fixation of the Bioreceptor	References
*Metal oxide NFs*				
ZnO (from PVP and ZnAc)	Thermal (700 °C). Addition of a PVA film	Gox (Glucose)	Adsorption	[35]
TiO_2_ (from PVP and Ti(BuO)_4_)	Thermal (500 °C, 5 min)	Gox (Glucose)	Adsorption followed by coverage with Chit membrane	[36]
TiO_2_ (from PVP and Ti(PrO)_4_) ^1^	Thermal (470 °C, 4 h). Oxygen plasma (COOH)	ChEt and ChOx (CholOl)	Covalent binding (EDC/NHS)	[37]
Mn_2_O_3_-Ag (from PVP, Mn(NO_3_)_2_, AgNO_3_)	Thermal (500 °C, 3 h). Dispersion of NFs in Nafion/GOx and casting	Gox (Glucose)	Entrapment into Nafion + cross-linking with GA	[38]
*Carbon NFs*				
Carbon-Cu (from PAN/PVP and CuAc_2_)	Thermal (280 °C, 2 h in air; 900 °C, 1 h in N_2_). Dispersion of NFs in Nafion/Lac and casting	Lac (catechol)	Entrapment into Nafion	[42]
Carbon-Ni (from PAN and NiAc_2_)	Thermal (280 °C, 2 h and 900 °C, 1 h in N_2_). Dispersion of NFs in Lac/DA and casting	Lac (catechol)	Entrapment into poly(DA)	[43]
Carbon-NCNS	Thermal (250 °C, 2 h in air; 900 °C, 30 min in N_2_)	Gox (Glucose)	Adsorption followed by coverage with Nafion membrane	[44]
Mesoporous carbon (from PAN and SiO_2_ NPs)	HF, 24 h Thermal	Gox (Glucose)	Adsorption	[45]
Carbon (from PAN)	Thermal (230 °C, 3 h in air; 300 °C, 2 h in H_2_/Ar; 1200 °C, 0.5 h in Ar); HNO_3_ 12 h (COOH); Growth of HA on NFs	CytC (H_2_0_2_)	Adsorption	[46]
Carbon (from PAN)	Thermal (230 °C, 3 h in air; 300 °C, 2 h in H_2_/Ar; 1200 °C, 0.5 h in Ar); HNO_3_ 12 h (COOH); Growth of PBNs on NFs	Gox (Glucose)	Entrapment in Chit membrane	[47]
*Polymer NFs*				
PMMA-MWCNTs(PDDA)		Gox (Glucose)	Adsorption	[48]
PANCAA-MWCNTs		Gox (Glucose)	Covalent binding (EDC/NHS)	[49]
Chit-MWCNTs (from Chit/PVA/MWCNTs)	0.5 M NaOH, 4 h (PVA removal)	Uricase (uric acid)	Cross-linking with GA	[50]
PAN/MWCNTs ^1^	Chemical reduction (LiAlH_4_)	PPO (catechol)	Cross-linking with GA	[51]
PAA/Nafion/Au NPs ^1^		HRP (H_2_O_2_)	Electrostatic interactions	[52]
PVA/AgNPs ^1^ (from PVA-AgNO_3_)	Reduction of AgNO_3_ with EGCG	HRP (H_2_O_2_)	Adsorption	[53]
PVA/PEI/AgNPs (from PVA/PEI)	Immersion in AgNO_3_ Reduction with EGCG	HRP (H_2_O_2_)	Adsorption	[53]
PVA/PEI-PdNPs (from PVA/PEI) ^1^	Immersion in PdCl_2_ and reduction with NaBH_4_	HRP (H_2_O_2_)	Adsorption	[54]
PAN-Au NPs-MWCNTs (from PAN)	Electrodeposition of AuNPs and electrophoretic deposition of MWCNTs	Gox (Glucose)	Covalent binding (EDC/NHS)	[55]
PAN/PANI ^1^	-	GDH (Glucose)	Covalent binding (EDC/NHS)	[56]
CA-CMC-PANI (from CA)	Immersion in CMC Immersion in ANI and polymerization	Lac (catechol)	Entrapment in Nafion	[57]
PLLA-PEDOT/PSS (from PLLA)	Immersion in EDOT-GOx and electropolymerization	Gox (Glucose)	Entrapment in PEDOT/PSS	[58]
Nylon 6,6-MWCNTs-poly(BIBA) (from Nylon 6,6-MWCNTs)	Immersion in BIBA and polymerization	Gox (Glucose)	Cross-linking with GA	[59]
PAN-MWCNTs/Ppy (from PAN_MWCNTs)	Immersion in FeTos and Py vapour polymerization	Gox (Glucose)	Adsorption	[60]
PS/Ir complex ^2^		Gox (Glucose)	Adsorption	[61]

^1^ Cyclic voltammetry detection; ^2^ luminescence detection, BIBA: 4-(4,7-di(thiophen-2-yl)-1H-benzo[d]imidazol-2-yl)benzaldehyde, CA: cellulose acetate, ChEt: cholinesterase, Chit: chitosan, CholOl: cholesterol oleate, ChOx: choline oxidase, CMC: carboxymethylcelulose, DA: dopamine, EGCG: epigallocatechin gallate, FeTos : Fe(III) p-tolueneslfonate, GDH: glucose dehydrogenase, GOx: glucose oxidase, HA: hydroxyapatite, HRP: horseradish peroxidase, Lac: laccase, MWCNTs: Multiwall carbon nanotubes, NCNS: nitrogen doped carbon nanosphere, PAN: polyacrylonitrile, PBNs: Prussian blue nanostructures, PDDA: poly(diallyldimethylammonium chloride), PEDOT: poly(3,4-ethylenedioxythiophene), PEI: polyethylenimine, PLLA: poly(L-lactide), PMMA: poly(methylmethacrylate), PPy: polypyrrole, PS: polystyrene, PSS: poly(sodium-p-styrene sulfonate), PVA: poly(vinylalcohol), PVP: polyvinylpyrrolidone.

**Table 2:** Enzymatic electrospun NFs-based biosensors for glucose detection.

Transduction	NFs/Transducer	Linear Range (mM)	LOD (µM)	Selectivity Test	Real Samples	References
*Immobilization of enzymes after electrospinning*						
Amperometry	l-Cys-GOx/PVA/ZnO NFs/Au electrode	0.25–19	1	Chol,l-Cys, AA, urea	No	[35]
	Chit/GOx/TiO_2_ NFs/Pt electrode	0.01–7	10	NR	No	[36]
	GOx-Nafion/Mn_2_O_3_-Ag NFs/GCE	up to 1.1	1.73	UA, AA	No	[38]
	Nafion/GOx/NCNS-ECNFs/GCE	0.012–1	2	UA, AA, DA	No	[44]
	GOx/Mesoporous ECNFs/SPE	Up to at least 20	NR	UA, sucrose	No	[45]
	GOx-Chit/PB/ECNFs/GCE	0.02–12	0.5	UA, AA, DA, mannose, galactose, fructose, lactate, BSA, l-Cys	No	[47]
	Nafion/GOx/MWCNTs(PDDA)-PMMA NFs/ITO	0.02–15	1	UA, AA	Human blood serum	[48]
	GOx/MWCNTs-PANCAA NFs /Pt electrode	0.67–7	670	NR	No	[49]
Amperometry	MWCNTs/Au NPs/PAN NFs/Au electrode	Up to 30	4	NR	No	[55]
	GOx-PEDOT-PSS/PLLA NFs/Pt microelectrodes	Up to 5 mM	120 (+700 mV)	NR	No	[58]
	GOx/PBIBA/MWCNTs-Nylon 6,6 NFs/GCE	0.01–2	9	AA, urea, oxalic acid	Beverages	[59]
	GOx/PPy/MWCNTs-PAN NFs/SPCE	0.125–7 (+0.36 mV)	980	NR	No	[60]
Luminescence	GOx/BSA/Ir complex-PS NFs	3 10^−7^–0.13	10^−4^	8 substances among which lactose, sucrose, fructose	Human blood serum	[61]
*Electrospinning of enzymes-polymer blends*						
Amperometry	GOx-PVA NFs/Au electrode	1–10	50	NR	No	[78]
	Nafion/GO-Chit-PVA NFs/Pt electrode	0.005–3.5	5	AA, UA, lactose, sucrose	Human blood serum	[80]
	GOx-Graphene-PVA NFs/Pt electrode	Up to 10	NR	NR	No	[81]
CV	GOx-MWCNT-PVASbQ NFs/Au electrode	0.005–4	2	NR	No	[82]
EIS	Au NPs/GOx-PVA-PEI NFs/Au electrode	0.01–0.2	0.9	AA, UA	No	[83]

AA: ascorbic acid, Chol: cholesterol, DA: dopamine, ECNFs: electrospun carbon nanofibers, GCE: glassy carbon electrode, GOx: glucose oxidase, ITO: indium tin oxide, MWCNTs: Multiwall carbon nanotubes, NR: not reported, PBIBA: poly-4-(4,7-di(thiophen-2-yl)-1*H*-benzo[d]imidazol-2-yl)benzaldehyde, PDDA: poly(diallyldimethylammonium chloride), PEDOT: poly(3,4-ethylenedioxythiophene), PLLA: poly(l-lactide), PMMA: poly(methylmethacrylate), PPy: polypyrrole, PS: poly(styrene), PSS: poly(sodium-*p*-styrene sulfonate), SPCE: screen-printed carbon electrode, SPE: screen-printed electrode, UA: uric acid.
